# Genome-Wide TSS Distribution in Three Related Clostridia with Normalized Capp-Switch Sequencing

**DOI:** 10.1128/spectrum.02288-21

**Published:** 2022-04-12

**Authors:** Rémi Hocq, Surabhi Jagtap, Magali Boutard, Andrew C. Tolonen, Laurent Duval, Aurélie Pirayre, Nicolas Lopes Ferreira, François Wasels

**Affiliations:** a IFP Energies Nouvelles, Rueil-Malmaison, France; b Génomique Métabolique, Genoscope, Institut François Jacob, CEA, CNRS, Université d’Evry, Université Paris-Saclay, Evry, France; University of Nebraska-Lincoln

**Keywords:** *Clostridium*, butanol, solvents, transcriptional regulation

## Abstract

Transcription initiation is a tightly regulated process that is crucial for many aspects of prokaryotic physiology. High-throughput transcription start site (TSS) mapping can shed light on global and local regulation of transcription initiation, which in turn may help us understand and predict microbial behavior. In this study, we used Capp-Switch sequencing to determine the TSS positions in the genomes of three model solventogenic clostridia: Clostridium acetobutylicum ATCC 824, C. beijerinckii DSM 6423, and C. beijerinckii NCIMB 8052. We first refined the approach by implementing a normalization pipeline accounting for gene expression, yielding a total of 12,114 mapped TSSs across the species. We further compared the distributions of these sites in the three strains. Results indicated similar distribution patterns at the genome scale, but also some sharp differences, such as for the butyryl-CoA synthesis operon, particularly when comparing C. acetobutylicum to the C. beijerinckii strains. Lastly, we found that promoter structure is generally poorly conserved between C. acetobutylicum and C. beijerinckii. A few conserved promoters across species are discussed, showing interesting examples of how TSS determination and comparison can improve our understanding of gene expression regulation at the transcript level.

**IMPORTANCE** Solventogenic clostridia have been employed in industry for more than a century, initially being used in the acetone-butanol-ethanol (ABE) fermentation process for acetone and butanol production. Interest in these bacteria has recently increased in the context of green chemistry and sustainable development. However, our current understanding of their genomes and physiology limits their optimal use as industrial solvent production platforms. The gene regulatory mechanisms of solventogenesis are still only partly understood, impeding efforts to increase rates and yields. Genome-wide mapping of transcription start sites (TSSs) for three model solventogenic *Clostridium* strains is an important step toward understanding mechanisms of gene regulation in these industrially important bacteria.

## INTRODUCTION

In bacteria, a key step in the regulation of gene expression is at the initiation of transcription, specifically whether and where the RNA polymerase binds DNA at the transcription start site (TSS) (1). In this regard, a precise description of the dynamics of TSS usage patterns can provide critical information for the comprehension of bacterial transcription regulation. Genome-wide determination of prokaryotic TSSs has been greatly facilitated by RNA-seq-derived methods that take advantage of the characteristic presence of a 5′ triphosphate on the initiating nucleotide of unprocessed RNAs (such as mRNAs). In the most recent approaches, these RNAs are selectively targeted by the vaccinia capping enzyme, which adds either a desthiobiotinylated (Cappable-seq) or a biotinylated (Capp-Switch seq) guanosine cap on triphosphorylated RNAs, permitting their capture on streptavidin-coupled beads (2–4).

In this study, we used the Capp-Switch sequencing methodology to map TSSs alongside the genomes of three clostridial strains: C. acetobutylicum ATCC 824, C. beijerinckii NCIMB 8052 and C. beijerinckii DSM 6423. These model solventogenic *Clostridium* strains share the capacity to ferment plant polysaccharides into a mixture of acetone, isopropanol, ethanol, and butanol (5). Despite this shared phenotypic trait, as well as others (anaerobic metabolism, ability to ferment a wide range of carbohydrates, sporulation), the C. beijerinckii and C. acetobutylicum species are clearly genetically distinct (6). Genetic differences have arisen even between the same-species strains NCIMB 8052 and DSM 6423, leading to distinct phenotypic characteristics and behavior (e.g., reduction of acetone) (7). These similarities and differences, arising from close but distinct evolutionary paths, are likely to be reflected at the genetic level. Comparing TSS distributions on the genomes of these strains could therefore highlight important gene regulation features, as functionally important TSSs theoretically have a high chance to be conserved.

We observed that Capp-Switch sequencing data sets are strongly biased toward the detection of TSSs for highly expressed genes, especially over 1,000 transcripts per kilobase million (TPM). We thus improved the Capp-Switch analysis pipeline by incorporating a normalization step based on RNA-seq expression. Using normalized Capp-Switch data sets, we described TSS distribution in these three model solventogenic strains, identifying primary and alternative TSSs at the genome-wide level. We experimentally validated our pipeline with the example of the central butyryl-CoA synthesis operon, for which secondary TSS positioning is species-specific. Finally, we identified conserved promoters across strains and compared the resulting maps, hypothesizing on the potential regulatory roles of these features and pinpointing how TSS identification can be used to access potentially crucial regulatory features which may differ between strains or species.

## RESULTS

### Manual TSS discovery in *C. acetobutylicum* from raw Capp-Switch data.

**FIG 1:**
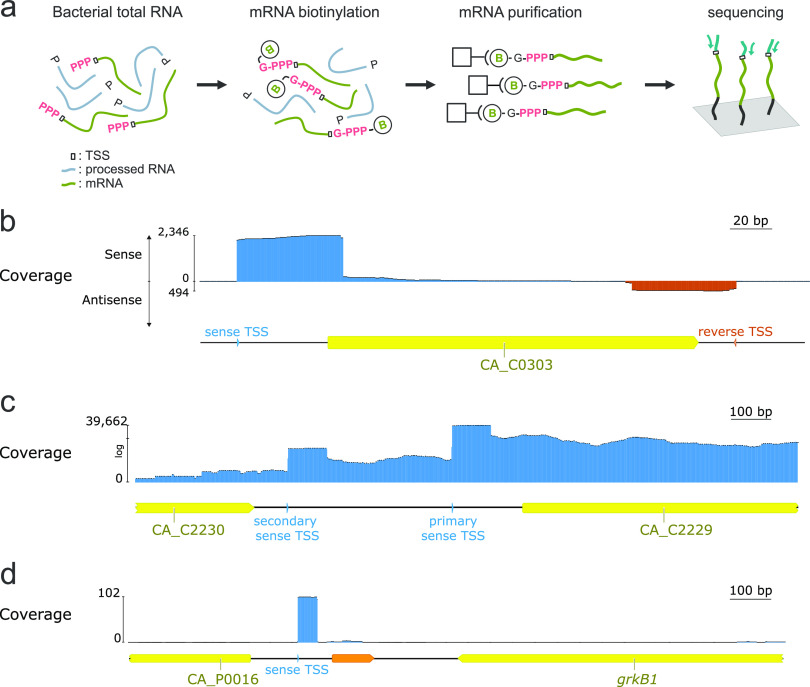
Capp-Switch library preparation protocol and manual data analysis. (a) Capp-Switch and RNA-seq libraries are constructed starting from isolated bacterial RNA. For Capp-Switch libraries, primary transcripts bearing a triphosphate at the 5′ end are first selectively purified using streptavidin beads. Both Capp-Switch and RNA-seq libraries were reverse transcribed using a template-switching enzyme and sequenced on the Illumina platform. (b to d) Raw Capp-Switch reads mapped on the genome of C. acetobutylicum ATCC 824. Predicted sense transcription start sites (TSSs) are shown as blue triangles, antisense TSSs are orange triangles. Predicted open reading frames are shown in yellow. (b) Opposing TSSs flank the ferredoxin gene CA_C0303. (c) Two alternative TSSs are located upstream of the pyruvate ferredoxin oxidoreductase gene CA_C2229. (d) Detection of a new coding sequence (orange) downstream from CA_P0016.

The Capp-Switch seq library preparation pipeline (Fig. 1a) (3) was applied to RNA samples extracted from mid-log-phase C. beijerinckii DSM 6423, NCIMB 8052, and C. acetobutylicum ATCC 824 cultures growing on glucose. C. acetobutylicum raw reads sequencing data were first examined manually using Geneious software (https://www.geneious.com) to identify TSSs along the chromosome and pSOL plasmid sequences. Although TSSs were easily identified in most cases, strong background signal, i.e., mapping of reads which apparently did not correspond to the 5′ ends of transcripts, hindered the precise identification of TSSs for some genes. In particular, highly expressed genes were covered by reads on their whole sequence, not only at the 5′ end of the transcript. This background noise was most likely detected because of nonspecific initiation of transcription or the TSS enrichment step retaining processed transcripts.

A total of 1,250 TSSs were manually identified, of 94.8% were purines (524 A and 661 G). This TSS mapping identified novel transcriptional features (Fig. 1b to d). For example, expression of the CA_C0303 gene, encoding a ferredoxin, is flanked by inward-facing TSS (Fig. 1b), suggesting that the antisense transcript could regulate CA_C0303 expression. As a second example, transcription of CA_C2229, encoding the pyruvate ferredoxin oxidoreductase, appears to be initiated at two upstream sites (Fig. 1c). It can be hypothesized that alternative TSSs are the result of transcription initiated by polymerases bound to alternative sigma factors, which might help regulate gene expression depending on specific conditions such as environmental stimuli or redox state. As a third example of how TSS data reveal novel transcript features, identification of a TSS at position 17,288 in the pSOL megaplasmid (Fig. 1d) lead to the identification of an unannotated coding sequence (8, 9). The product of this gene is identical to holin-like toxins identified in the *Clostridium* genus and shares 41.2% identity with the antibacterial protein Tmp1 (10).

### Expression normalization improves TSS detection.

We found that genes which are highly expressed in published RNA-seq data sets (7, 11) often had multiple TSSs. Hence, we reasoned that the final threshold set on reads per million (RPM) values of TSSs (i.e., 10 RPM for Capp-Switch sequencing [3]) needed to be dynamically adjusted to consider the local gene expression downstream from each TSS. Each TSS is associated with a single gene based on its localization (intragenic, positioned inside the associated gene; intergenic, positioned outside the associated gene, defined as having the closest gene start or end relative to the TSS). We performed RNA-seq on the same RNA samples that were used to prepare Capp-Switch seq libraries. We then normalized the strength of each TSS (in RPM) with the RNA-seq expression (in transcripts per kilobase million [TPM]) of its associated gene (Fig. S1 in the supplemental material). Because each identified TSS is associated with a normalized RPM value which is dependent on gene expression, this method allowed us to retain, in practice, an invariable, simple, normalized RPM cutoff at the end of the data analysis pipeline (10 normalized RPM, or 25 for a higher confidence).

**FIG 2:**
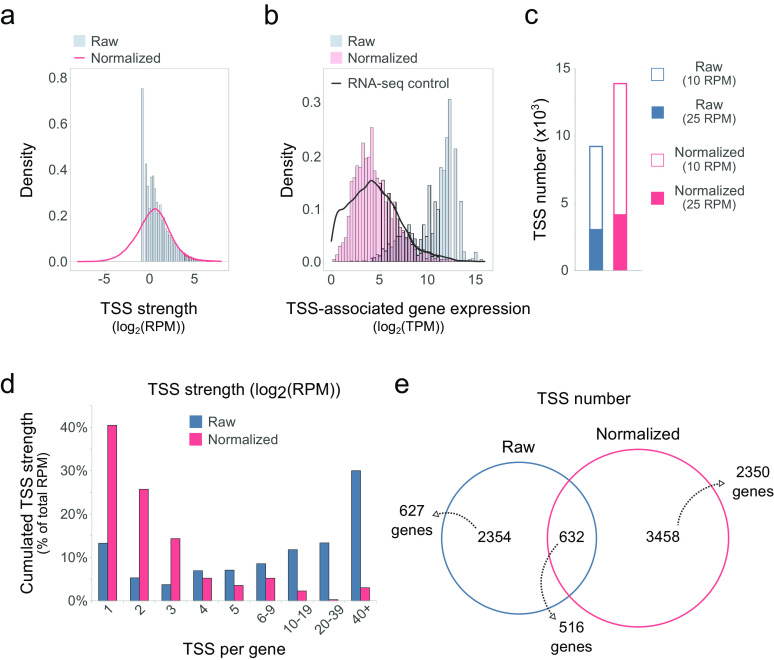
Normalization of Capp-Switch reads enhances TSS identification for C. beijerinckii DSM 6423. (a) TSS expression distribution with (*normalized*) and without (*raw*) expression normalization (10 reads per million [RPM] [TSS] detection threshold shown). (b) RNA-seq expression distributions of (i) all genes (black line) and (ii) the subset of genes for which TSSs have been found, in *normalized* (blue) and *raw* (red) data sets (25 RPM detection threshold shown). (c) Number of TSSs found for *normalized* and *raw* data sets. (d) TSS number per gene (with detected TSSs, 25 RPM detection threshold shown). Value corresponds to the number of reads falling in each category (as percentage of the total RPM). (e) Venn diagram showing TSSs found for each data set (25 RPM detection threshold shown) and the corresponding numbers of associated genes.

Next, we compared the data sets for each strain, with (*normalized*) and without (*raw*) normalization (Fig. 2, Fig. S2 and 3). Adjusting TSS strength relative to local gene expression significantly changed the data set distribution (Fig. 2a, Fig. S2). When we compared the distribution of the expression of gene subsets with a detected TSS, this further resulted in a significant shift from highly expressed genes (*raw* data set; >1,000 TPM) to a distribution representative of the whole RNA-seq data set (Fig. 2b). This observation suggests that our novel analysis detected TSSs more evenly along the bacterial genome. Normalization also resulted in a greater number of TSSs (Fig. 2c), which probably resulted from the higher sensitivity when additional data were considered.

We subsequently tested our hypothesis that in the original data set, TSSs tend to accumulate on a few genes (Fig. 2d). A high proportion of reads (>70%) contributed to the detection of TSSs in genes that bore more than 4 TSSs, with a maximum of 244 TSSs for a single gene (Fig. S3). Conversely, expression normalization reduced the proportion of genes with more than 4 TSS to 15%, indicating that (i) that a higher number of genes overall were found with one or several TSSs and (ii) the normalization step improved the data set by removing secondary TSSs which were linked either to pervasive transcription or to methodological noise.

Finally, we compared TSSs in both data sets (Fig. 2e). Most were data set-specific, which underlines the impact of an additional normalization step on TSS identification.

To summarize, optimized expression normalization allows an even detection of TSSs along the bacterial genome. Indeed, *raw* data sets tend to identify TSSs on very highly expressed genes, which is detrimental because they only represent a fraction of expressed genes. Our normalization pipeline enhances TSS detection in an expression-independent manner and increases the number of genes with detected TSSs.

### Capp-Switch identifies thousands of TSSs in 3 clostridial genomes.

**FIG 3:**
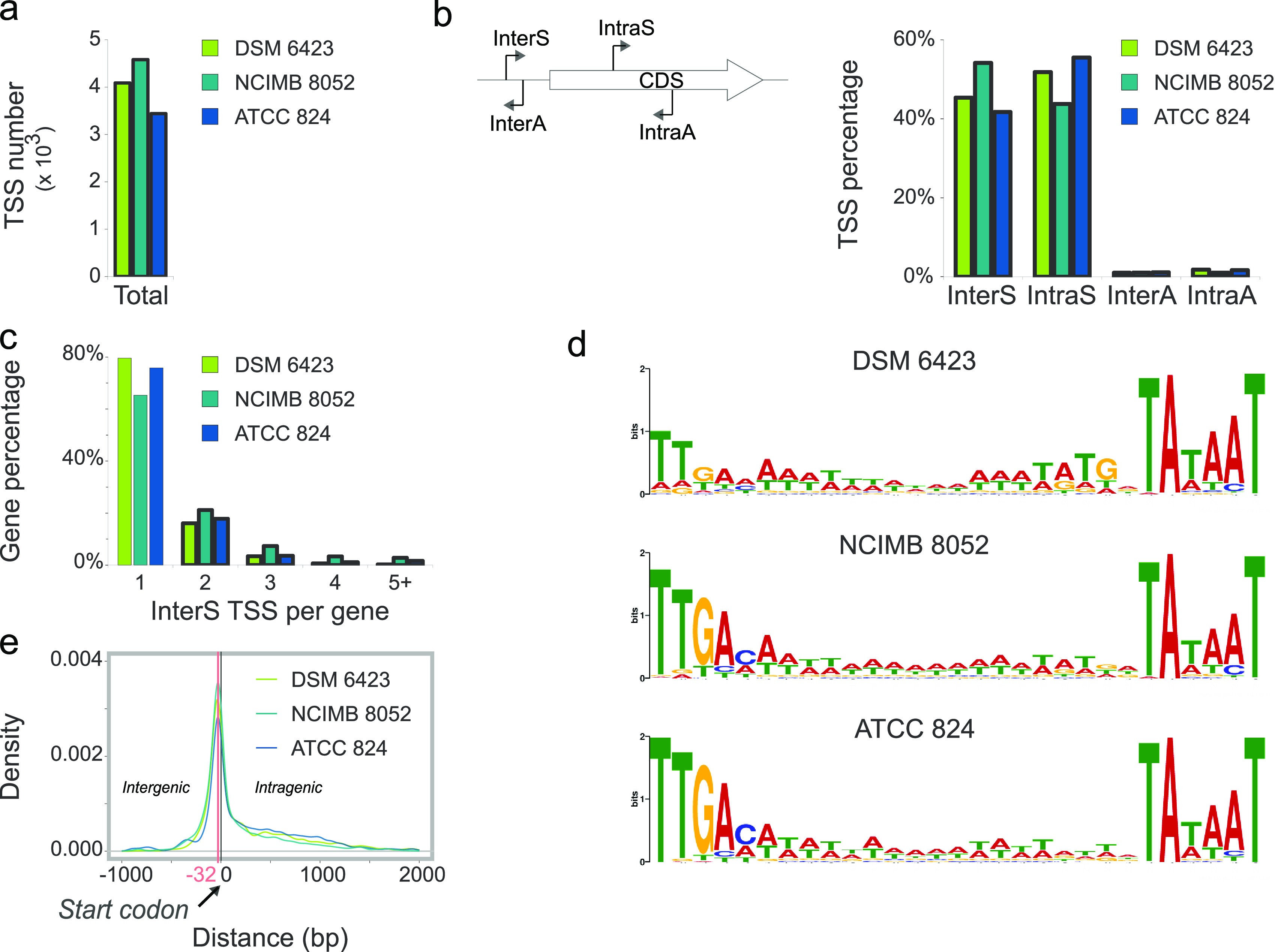
General transcriptomic features of C. beijerinckii DSM 6423, C. beijerinckii NCIMB 8052, and C. acetobutylicum ATCC 824. (a) Number of TSSs found for each strain with a confidence threshold of 25 RPM. (b) Classification of TSSs in 4 categories: intergenic sense (InterS), intragenic sense (IntraS), intergenic antisense (InterA), and intragenic antisense (IntraA). (c) Number of InterS TSSs per gene for each strain. Values correspond to the percentage of genes with detected TSSs. (d) The −35 and −10 motifs found upstream from InterS TSSs of the three strains (e). 5′ UTR length distributions, calculated as the distance between an InterS TSS and coding DNA sequence (CDS) starts.

After optimizing the TSS identification pipeline, we focused on the resulting data for each strain (Fig. 3, Table S1, and Fig. S4). Analysis of duplicate cultures permitted the detection of 4,090, 4,583, and 3,441 TSSs in C. beijerinckii DSM 6423, NCIMB 8052, and C. acetobutylicum ATCC 824, respectively, with a high confidence threshold (>25 normalized RPM, Fig. 3a). Adjusting the threshold to 10 normalized RPM increased the number of TSSs to over 13,000 and 9,000 for the C. beijerinckii and C. acetobutylicum strains, respectively. TSSs identified in C. acetobutylicum were compared to previously experimentally validated TSSs for 11 genes (with a primer extension or 5′ RACE; Table S2). All previously identified TSSs were found in the normalized 10 RPM data set, and in 9 of 11 genes in the 25 normalized RPM data set. However, the latter data set limits the discovery of novel, likely pervasive, lowly expressed secondary TSSs. Hence, we only used the 25 normalized RPM data sets in subsequent analyses to limit the false discovery rate.

TSSs were classified in 4 categories depending on their orientation and localization relative to the associated genes: InterS (intergenic TSS with downstream gene in same orientation), InterA (intergenic TSS with downstream gene opposite orientation), IntraS (intragenic TSS in gene with same orientation), or IntraA (intragenic TSS in gene with opposite orientation) (Fig. 3b). In the 3 strains, TSS repartition was relatively similar, with most TSSs identified in the sense direction (InterS: 40 to 55%; IntraS: 40 to 55%). Such an abundance of intragenic TSSs has been observed on several occasions using different methodologies (4, 12), and this has been hypothesized to mainly be the result of pervasive transcription, in some cases, however, with a conserved function (such as driving the expression of truncated proteins or ncRNAs). This high number of IntraS TSSs, however, must be considered in light of the high proportion of coding sequences in bacterial genomes (88%, 81%, and 83% of the ATCC 824, NCIMB 8052, and DSM 6423 genomes, respectively). Even though the numbers of InterS and IntraS TSSs were similar, most of the reads contributed to InterS TSSs (≈56 to 65% of total reads, Table S3). These canonical, intergenic TSSs were found upstream from 1,468 genes in DSM 6423 (23% of genes), 1,525 genes in NCIMB 8052 (29%), and 976 genes in ATCC 824 (25%). For most of these genes, our data further revealed that, in our experimental conditions, transcription was controlled by a single InterS TSS (in 65 to 80% of the cases, depending on the strain; Fig. 3c). As expected, conserved −10 and −35 motifs were found enriched upstream from detected TSSs in all three strains (Fig. 3d), confirming these were *bona fide* TSSs. Less than 3% of the TSSs were observed in the antisense direction, which supports previous results obtained for *C. phytofermentans* (3) (Fig. 3b). In accordance with this study, we observed that these antisense transcripts may have important biological functions (Table S4). Indeed, antisense transcription initiation events were often detected for genes involved in transcriptional control, redox control, and sugar uptake, suggesting that antisense transcription may regulate some of these important cellular processes *in vivo*. Confirmation of antisense transcription from some InterA and IntraA TSS was achieved by mapping visualization of forward reads from paired-read RNAseq (Fig. S5).

The 5′ UTR lengths (measured as distances in bp between InterS TSS positions and corresponding coding DNA sequence [CDS] starts) rarely exceeded 200 bp in the three strains (Fig. 3e), exhibiting no correlation with gene expression (Data Set S8). Most 5′ UTRs were between 0 and 100 bp long (with a peak at −32 bp relative to the start codon), with ≈2 to 3% of transcripts categorized as leaderless (transcripts not bearing an upstream RBS; for this analysis, 5′ UTR length of <6 bp), suggesting that, despite being common in other bacteria (13, 14), leaderless transcription seems to occur at relatively low levels in solventogenic clostridia.

Alternative sigma factor binding sites were also identified in the sequences upstream from InterS TSSs using RSAT (15) and a collection of motifs described in the literature (Table S5). Only a very small subset of genes is transcribed via alternative sigma factors in the three strains, which is not surprising, as alternative sigma factor-based regulation has mostly been shown to regulate sporulation with regulons typically constituted by a few genes (16). Expression using alternative sigma factors may be more common under non-ideal growth conditions.

### Capp-Switch reveals that acetoacetyl-CoA conversion is differentially controlled in *C. acetobutylicum* and *C. beijerinckii*.

**FIG 4:**
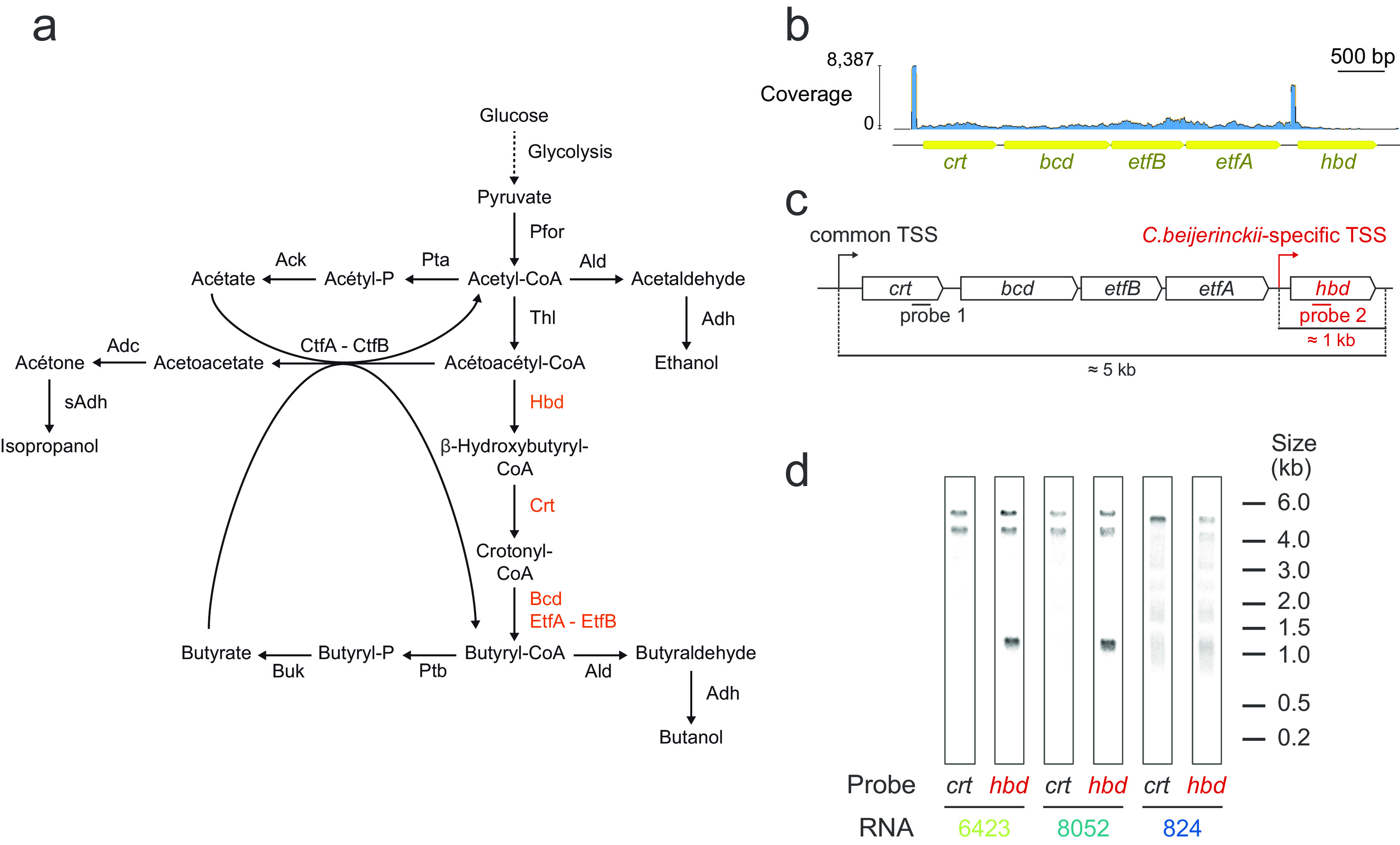
Northern blot analysis of the butyryl-CoA synthesis (BCS) transcript structures. (a) Metabolic pathways orienting solventogenic metabolisms toward acetone/isopropanol or butanol formation. Pfor, pyruvate:ferredoxin oxidoreductase; Thl, thiolase; Hbd, β-hydoxybutyryl-CoA dehydrogenase; Crt, crotonase; Bcd, butyryl-CoA dehydrogenase; Etf, electron transfer flavoprotein; Pta, phosphotransacetylase; Ack, acetate kinase; Ptb, phosphate butyryltransferase; Buk, butyrate kinase; Ctf, acetoacetyl-CoA–acetate/butyrate-CoA transferase; Adc, acetoacetate decarboxylase; Ald, aldehyde dehydrogenase; Adh, alcohol dehydrogenase; sAdh, secondary alcohol dehydrogenase. (b) Raw Capp-Switch reads mapped on the genome of C. beijerinckii NCIMB 8052. Annotated open reading frames are represented in yellow. (c) Hybridization sites of the probes used for Northern blotting are highlighted for the *crt* and *hbd* genes. (d) Anti-*crt* and anti-*hbd* Northern blotting was performed for each strain.

Solventogenic clostridia have an atypical metabolism that allows them to convert multiple carbon sources to a panel of high-interest industrial compounds (i.e., butanol, ethanol, acetone, isopropanol, 2,3-butanediol). These metabolic pathways are centered around acetyl-CoA (Fig. 4a), which serves as the fundamental building block for these metabolites. To synthesize acetone or isopropanol and butanol, two molecules of acetyl-CoA are first condensed into one acetoacetyl-CoA, which is transformed into acetone/isopropanol via the CtfAB-Adc-Sadh route, or into butanol by enzymes encoded by the butyryl-CoA synthesis (BCS) operon and subsequently by aldehyde/alcohol dehydrogenases.

The BCS pathway involves the products of 5 genes (*hbd*, *crt*, *bcd*, *etfA*, *etfB*) and was described several decades ago as a single operon in C. acetobutylicum (17). Even though previous transcriptomics analyses suggested that this organization was conserved in both C. beijerinckii strains (7, 11), our comparative TSS analysis revealed that, in addition to the TSS located upstream of the first gene (*crt*) in all three strains, there was a novel and highly used TSS in C. beijerinckii strains located upstream from the *hbd* gene (i.e., the last gene) coding for the first enzyme involved in the metabolic pathway (Fig. 4b).

These results were experimentally verified by Northern blotting (Fig. 4c and d). For each strain, single-stranded radiolabeled DNA probes targeting either *crt* or *hbd* were hybridized to nitrocellulose-transferred bacterial RNAs. The results show an *hbd* transcript (≈1 kb) specific to C. beijerinckii strains. Anti-*hbd* Northern blots also revealed a high-molecular weight signal (between 4 and 6 kb) similar to anti-*crt* Northern blots, suggesting that *hbd* is also transcribed as a part of the original BCS operon. However, this signal surprisingly constitutes two distinct transcripts, detected in both C. beijerinckii assays. These transcripts, therefore, contain both the anti-*crt* and anti-*hbd* probe binding sites, suggesting either the existence of an upstream, lowly expressed TSS which was not detected by our Capp-Switch approach, or a form of transcript processing (such as the ones described by Gill et al. [18]) which precisely shortened some of the BCS transcripts.

To summarize, an experimental approach allowed us to check the biological relevance of our Capp-Switch data, which indicated the presence of an alternative TSS for *hbd* transcription in C. beijerinckii. Northern Blot analysis confirmed its presence, and further indicated an operonic/sub-operonic structure driving *hbd* expression.

### Promoter comparison across strains.

**FIG 5:**
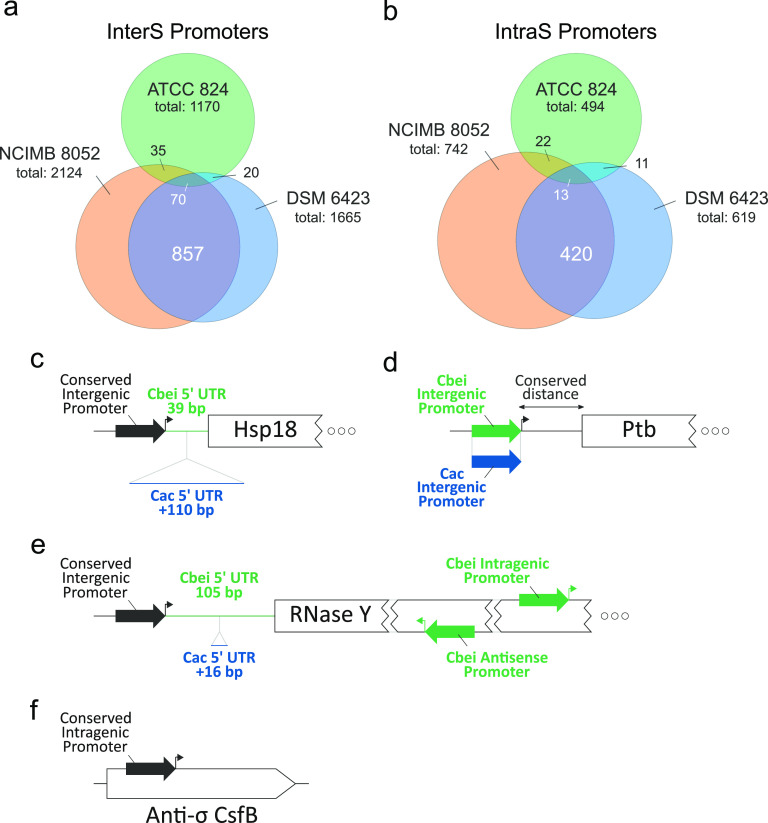
Promoter comparison in the three strains. (a and b) Venn diagrams showing the number of (a) InterS and (b) IntraS TSSs conserved and not conserved after ortholog pair identification, promoter alignment, and threshold-based selection. (c to f) Selected examples of TSSs with or without conservation in the three strains.

Promoters are likely to be conserved across close species if they are under selective pressure (12). With this in mind, we compared promoter sequences in the three bacterial genomes, focusing on InterS TSSs (filtered so that the distance between the TSS and the start codon was less than 200 bp) and IntraS TSSs (Fig. S7). For each strain, promoter sequences (50 bp upstream from TSSs) were extracted and aligned using pairwise alignments. To do this, promoter sequences associated with homologous genes were aligned (homology  > 60%). An alignment score threshold was chosen for each pair of strains based on the distribution of alignment scores. This pipeline allowed the recovery of 1,396 and 691 conserved InterS and IntraS promoters, respectively (Table S6). Alignments were further filtered so that each promoter was only associated with a single promoter from another strain (Fig. 5a and b). Most strikingly, the number of associated C. beijerinckii*-*C. acetobutylicum promoters was very low (≈10% of C. acetobutylicum promoters from genes which have orthologs in C. beijerinckii were conserved in at least one of the C. beijerinckii strains), underlining a poor conservation of promoters between these species. Despite their phenotypical resemblance, these organisms might have diverged a sufficiently long time ago (as suggested in a recent phylogenetical evaluation [6]), implying that promoter sequences cannot be associated using our approach. On the other hand, comparison of the two C. beijerinckii strains using our pipeline indicated that about half of InterS and a third of IntraS promoters were conserved. Therefore, we performed functional enrichment of genes with promoters conserved for the two C. beijerinckii strains (Fig. S8). While some categories appear slightly depleted or enriched, the distribution of gene categories with conserved promoters seems relatively similar to the gene category distribution of the genome, suggesting that gene function is not highly relevant to the conservation of the considered promoters.

We subsequently looked at promoter conservation at the gene level and found some interesting examples (Fig. 5c to f). Hsp18 is an important heat shock protein in solventogenic clostridia, as it is induced at the onset of solventogenesis and presumably involved in solvent resistance (19–21). The corresponding promoter is well conserved in all three strains, but the 5′ UTR lengths are different (C. acetobutylicum, 149 bp, as previously reported [19]; C. beijerinckii, 39 bp, Fig. 5c). This could be linked to additional post-transcriptional gene regulation in C. acetobutylicum. Indeed, the 149-bp 5′ UTR from C. acetobutylicum reveals an extensive secondary structure (as shown by using RNAfold with default parameters [22]), which could regulate transcript translation via a riboswitch-like mechanism, i.e., by promoting premature transcription termination or inhibiting translation initiation. In contrast, the 5′ UTR length of *ptb*, a central metabolic gene involved in butyrate formation and solventogenesis regulation (23, 24), is strictly the same among species, but the promoters are species-specific, suggesting that a differential transcriptional regulation exists for these genes in the two species.

Because of their putative regulatory role, intragenic promoters are interesting to analyze, since strong conservation might imply a relevant functional role. In the case of the RNase Y gene, which codes for an essential protein involved in mRNA decay (25), the canonical promoter was well conserved in all three strains, with similar 5′ UTR lengths (Fig. 5e). However, in both C. beijerinckii strains, very strong internal promoters were also detected. In particular, an antisense promoter strongly conserved in these two strains might repress RNase Y expression via a RNA polymerase collision mechanism (26) (supported by strand-specific RNA-seq reads; Fig. S5A). For the anti-sigma factor CsfB, an inhibitor of the sporulation- and solventogenesis-related σ^G^ and σ^E^ factors (27), an especially strong intragenic promoter is conserved in all three strains (Fig. 5f). Hence, this promoter might be involved in the regulation of this gene in the three strains, and therefore in the regulation of solvent production and spore formation.

As illustrated by these few examples, comparing promoter conservation across species can highlight differential or similar transcriptional features, discovering potentially important information on how various processes (such as solventogenesis) are regulated.

## DISCUSSION

In this work, we investigated transcription initiation by determining TSSs in three related solventogenic clostridia. In this regard, Capp-Switch sequencing (3) was used and first refined to include an optimized normalization pipeline, in an attempt to better reflect the biological relevance of identified TSSs. To do this, gene expression data analysis was incorporated into the detection pipeline by performing RNA-seq on the same mRNA samples and using the resulting expression values to normalize Capp-Switch seq data. This additional step limited the gene expression bias and hence enhanced results at the genome scale (expression bias, TSS number/gene) and the gene scale (Fig. S3), underlining the importance of treating Capp-Switch data with a normalization step.

This improvement could be due to biological and/or technical causes, e.g., if strong promoters increased permissive transcription events around the vicinity of the primary TSS or if the purification technique was not fully efficient. Indeed, for highly expressed genes, even a small proportion of co-purified undesirable RNAs (i.e., fragmented RNAs for which the 5′ end does not correspond to the TSS) could bias the TSS detection pipeline. In both cases, accounting for expression helps to mitigate this problem and detect *bona fide* TSSs, particularly for low or moderately expressed genes.

These results therefore highlight the necessity of a second sequencing to treat Capp-Switch seq data. In their original work, Ettwiller and colleagues (4) previously questioned the necessity of a control sequencing for Cappable-Seq (in this case, a library where the triphosphate (PPP) 5′ end purification step is omitted), similarly to what had been done until then for dRNA-seq (2). However, they concluded that this control was unnecessary under their conditions because it only allowed the elimination of a minority of TSSs. These discrepancies with our conclusions might come from technical differences (i.e., the way RNAs are processed in Capp-Switch seq versus Cappable-seq) or the way TSS data were compared to the control in both experiments. Indeed, local enrichment was considered for Cappable-seq, whereas in Capp-Switch seq we accounted the overall expression of the associated gene for each TSS. This might considerably change the observations, as Illumina RNA-seq produces uneven coverage along genes; in particular, lower coverage at the 5′ end (28, 29).

Our genome and gene analyses (Fig. 3, Fig. S3) indicated that, for the three strains, Capp-Switch accurately detects TSSs at the single-nucleotide resolution. Importantly, TSSs have similar features in the three clostridia (total and pro-gene number of TSSs, TSS categories, 5′ UTR length, upstream motifs). One interesting feature is the very low number of antisense TSSs, which was already found in *C. phytofermentans* using the same method (3). Indeed, it is well known that, in clostridia, antisense transcription exists and has a relevant biological role (30, 31). Our data, however, suggest that antisense transcription initiation is uncommon in these organisms and might be restricted to a few genes or linked to very weak promoters, making its detection uncertain when using the same pipeline used for sense TSSs.

While we observed many inter-strain TSS similarities at the genome-wide scale, there were interesting differences at the gene scale. In particular, the BCS operon is a striking example of how TSS data can be readily used to spot differences between microorganisms that would otherwise be difficult to distinguish/discriminate using RNA-seq only. Indeed, for both C. beijerinckii strains, previous transcriptomics studies predicted the same gene organization as for C. acetobutylicum (7, 11). However, our data show that C. beijerinckii uses an operonic/sub-operonic structure for the expression of BCS genes, with *hbd* under the control of its own promoter. Given that Hbd catalyzes the initial step of the metabolic pathway, separate transcription regulation could allow tighter control of the whole pathway, possibly to fine-tune the balance of acetoacetyl-CoA conversion into butanol or into acetone/isopropanol.

This interesting interspecies difference prompted us to develop a pipeline to automatically retrieve conserved promoters between the three strains. Intuitively, promoters under strong selective pressure are more likely to be conserved as being highly functional. Conversely, non-conserved promoters may reflect weaker selective pressure or a different evolutionary path of the compared organisms. This approach is similar to the one Shao et al. adopted (12), which identified secondary intragenic TSSs as a highly conserved feature in various *Shewanella* species. Our results, however, indicate that promoters diverged sufficiently between C. acetobutylicum and C. beijerinckii so that only a few of them were conserved. However, these promoters most likely drive crucial functions (see examples from Fig. 5). Between both C. beijerinckii strains, intergenic and intragenic promoter conservation is, as expected, much higher. Conservation of these promoters, however, does not seem to be related to gene function (Fig. S8), which is surprising given our initial hypotheses. This observation could be explained by the fact that crucial functions are distributed over various categories, or that tight promoter control might only be necessary in some instances, not at the gene category level.

Comparison of promoters between the three strains also has the potential to shed light on unannotated genes and regulatory pathways. For example, this is the case for CsfB, an anti-sigma factor targeting sporulation-specific sigma factors which has only been studied in B. subtilis (26) but could also have a highly relevant biological function in solventogenic clostridia. Indeed, the regulation of sporulation-specific sigma factors (via transcription interference, as suggested by our data for *csfB*) is likely to have a strong impact on solventogenesis, since both aspects are intertwined (32–35). This is especially important because, even though alcohol production is the major reason for the study of solventogenic clostridia, the solventogenesis regulatory circuitry is still only partly understood (36).

Overall, new aspects revealed by our TSS-mapping data can complete previous transcriptomic studies of solventogenic clostridia. Making these maps available to the community will undoubtedly further our comprehension of gene expression and help formulate relevant metabolic engineering strategies for these industrially relevant microorganisms.

## MATERIALS AND METHODS

### Strains, media, and culture conditions.

Clostridial strains (ATCC 824, NCIMB 8052, DSM 6423) were grown anaerobically at 34°C in liquid 2YTG (16 g tryptone, 10 g yeast extract, 5 g NaCl, and 20 g glucose per L [pH 5.2]). Solid medium was prepared with 15 g/L agar and 5 g/L glucose, without pH adjustment. For RNA isolation, 10 mL of mid-log-phase duplicate cultures (optical density at 600 nm ≈ 0.5) was harvested and stabilized on ice with 1.25 mL cold equilibration solution (a 1:18 proportion of acid phenol:ethanol). After centrifugation, cell pellets were kept at −80°C until further use.

### RNA extraction.

Frozen pellets were suspended in TRIzol reagent (Invitrogen). Following cell lysis, chloroform was added, and the aqueous phase was isopropanol-precipitated. Total RNA was then treated with Turbo DNase (Invitrogen) and further purified (RNA Clean and Concentrator-5, >200-bp protocol, Zymo Research). Ribosomal RNAs were depleted using the RiboZero kit (Illumina) and the resulting RNAs were stored at −80°C until further use.

### RNA-seq library preparation.

RNA-seq libraries were prepared with a template-switching protocol. After depletion of rRNA (RiboZero, Illumina), Moloney murine leukemia virus (MMLV) reverse transcriptase (SMARTScribe, Takara Bio) was used to obtain cDNAs with the SMART Stranded N6 Primer mix (Takara Bio). cDNAs were purified with AMPure magnetic beads (Beckman Coulter). Libraries were next obtained by PCR using beads as the template (SeqAmp polymerase, Universal Forward and Indexed Reverse primers, Takara Bio). After on-bead purification, libraries were sequenced with a MiSeq device (Illumina).

### Capp-Switch library preparation.

The Capp-switch sequencing library preparation protocol was used (3). Briefly, a 5′ biotinylated cap was first added to the 5′-PPP RNAs using vaccinia capping enzyme (New England Biolabs). RNAs were fragmented and 5′-capped RNAs were enriched using streptavidin beads. cDNA synthesis was performed directly on the beads with the template-switching method, using the SMARTscribe MMLV RT (Clontech). Eluted cDNAs were further used as templates for PCR (Universal Forward PCR primer and Indexed Reverse PCR primer, Clontech Laboratories), and the resulting libraries were sequenced on an Illumina MiSeq.

### Accession number(s).

Sequencing reads from RNA-seq and Capp-Switch seq have been submitted to the SRA Database (BioProject accession no. PRJNA767822).

### Capp-Switch data treatment pipeline.

Capp-Switch forward reads were trimmed to remove the 3-bp reverse transcriptase extension derived from the template switching library preparation protocol. Capp-Switch and RNA-seq reads were then mapped to the relevant genomes (C. acetobutylicum ATCC 824, GCA_000008765.1; C. beijerinckii NCIMB 8052, GCA_000016965.1; C. beijerinckii DSM 6423, GCA_900010805.1) using Geneious R10. 95 to 99% of reads were mapped to unique positions, yielding between 0.73 million (rep. 2 ATCC 824 Capp-Switch) and 2.9 million (rep. 1 NCIMB 8052 RNA-seq) reads per sample (Table S6). Capp-Switch data were then treated with a succession of custom Perl scripts (3). Briefly, TSSs were first identified by counting the number of forward reads, starting at each genomic position. Genome annotations were used to associate TSSs with the closest genes and classify these TSSs in four subcategories (3) (InterS, InterA, IntraS, or IntraA, see Results). TSS replicates were compared, and positions not detected in both duplicates were discarded. Next, gene annotations were used to associate TSS and RNA-seq data sets. Normalized data sets were obtained by dividing TSS raw read counts by the mean RNA-seq expression values (in TPM) of TSS-associated genes and scaling the resulting values back to a total of 1 million reads. In the case of the ATCC 824 strain, only one RNA-seq could be performed as no RNA for replicate 1 was left after the initial round of Capp-Switch sequencing. In this case, the TPM value of replicate 2 was used for normalization. TSS positions were next clustered in the *raw* and *normalized* data sets by retaining positions with the highest numbers of reads in 5-bp sliding windows. These positions were further filtered out with a cutoff of 10 or 25 (high-confidence data set) read starts per million reads.

### Motif analysis.

The InterS subcategory of TSS was considered for motif analysis. The 50 bp upstream from each InterS TSS were extracted from the genome sequences of the 3 strains and defined as promoter sequences. These were fed into MEME (37) for motif discovery. All parameter settings were kept at default and statistically significant motifs were selected based on their E values (<0.05).

### Detection of TSSs conserved in the different strains.

The overall illustration of the pipeline is shown in Fig. S7 in the supplemental material. To detect conserved promoters in the 3 strains, we first filtered the TSS subcategory InterS based on the distance (*d* < 200 bp) between the TSSs and their associated gene starts. The promoter sequences for the InterS and IntraS categories were extracted 50 bp upstream of mapped TSSs. We performed all possible combinations of pairwise alignments, using the Needleman-Wunsch algorithm, across all strains for the respective subcategories. Instead of giving an empirical threshold of the alignment score to select conserved TSS, orthology-driven mapping of promoters was considered. Orthology information was obtained from the MicroScope database (38). For each pair of strains, we split the alignment scores into two groups (with alignment scores of promoters whose genes were orthologs or non-orthologs). For these two lists of alignment scores, we observed that the probability distribution for the scores of the orthologs group was different from that of the non-orthologs group. For each pair of strains, we selected an alignment score threshold based on how these distributions overlapped. For RNase Y, the antisense promoter was manually spotted and aligned for the 3 strains.

### Study of specific sigma factors.

Based on a list of 13 motifs related to different DNA-binding regulators (Sigma A, Sigma 54, Sigma E, Sigma F, Sigma G, Sigma H, Sigma K, and Spo0A), we investigated, for each strain, whether these motifs were recovered in the InterS promoter set (Table S5).

To this end, we applied the *dna-pattern* tool provided in the pattern-matching suite of RSAT (14) to all promoter sequences for each strain. We parametrized the algorithm to authorize degenerated bases based on the IUPAC code, as well as indels in the pattern. A motif search was performed on both strands and overlapping of successive matches was prevented. For each motif, we obtained for each promoter the number of matches and their positions. We then post-treated this information to obtain statistics on the presence of specific sigma factors on the InterS promoters of the three strains independently.

### Northern blotting.

For each lane, 10 μg heat-denatured total RNA was size-separated in a denaturing 1.2% agarose gel (formaldehyde 6.6%). Following electrophoresis, RNAs were transferred overnight by capillarity with 10 × SSC buffer (1×: 150 mM NaCl, 15 mM sodium citrate [pH 7.0]) onto a nitrocellulose membrane (Hybond N+, GE Healthcare). Probes were obtained in two steps. First, ≈300- to 400-bp fragments of target genes were amplified by PCR on genomic DNA. Second, these PCR products were used as templates for unidirectional PCR (≈250- to 350-bp amplification of reverse strands, with incorporation of α-^32^P dATP; Perkin Elmer). The primers used for these PCRs are shown in Table S8. RNAs were UV-cross-linked onto the membranes and pre-blocked for 1 h at 65°C with salmon sperm DNA in Church buffer (0.5 M sodium phosphate [pH 7.0], 7% SDS, 1 mM EDTA). Radioactive probes were subsequently added for hybridization for 6 h at 65°C. After two washes in 2 × SSC 0.5% SDS buffer and one wash in 0.1 × SSC 0.5% SDS buffer at 65°C, radioactivity was revealed by exposition on a phosphor screen (GE Healthcare) and analysis of this screen using a Typhoon imager.
