# Supplementary material for: Genome-Wide TSS Distribution in Three Related Clostridia with Normalized Capp-Switch Sequencing
Source: Microbiol Spectr. 2022 Apr 12;10(2):e02288-21. doi: 10.1128/spectrum.02288-21 (PMC9045289; doi:10.1128/spectrum.02288-21)
Supplement: SUPPLEMENTAL FILE 9 — Fig. S1 to S8. Download SPECTRUM02288-21-s009.pdf, PDF file, 1 MB [file spectrum02288-21-s009.pdf]

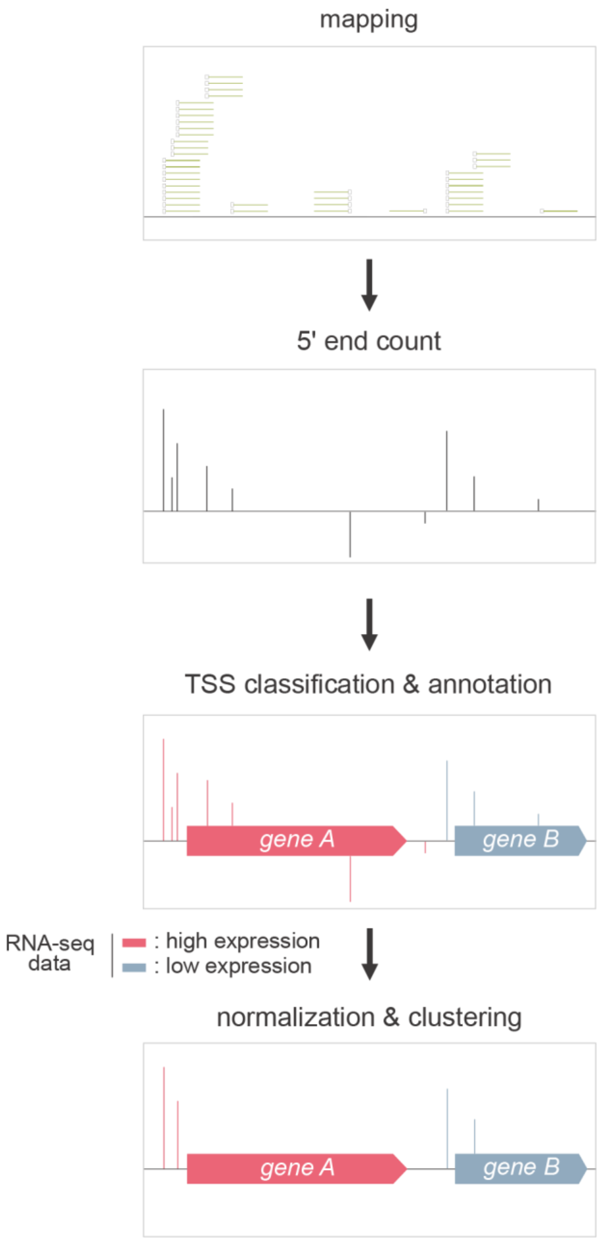

Supplementary Fig S1: **Capp-Switch seq data treatment pipeline.** Trimmed reads are first mapped against the reference genome. For each genome position, 5’ ends are counted in sense or reverse orientation to determine TSS position and strength. TSSs are next associated with their closest genes and classified in 4 categories according to their orientation (sense, antisense) and their position (intragenic, intergenic) relatively to the associated genes. These data are used to annotate each TSS. RNA-seq TPM count is next used to normalize TSS strength. Finally, TSSs in 5 bp sliding windows are clustered together, retaining the position of the strongest TSS.

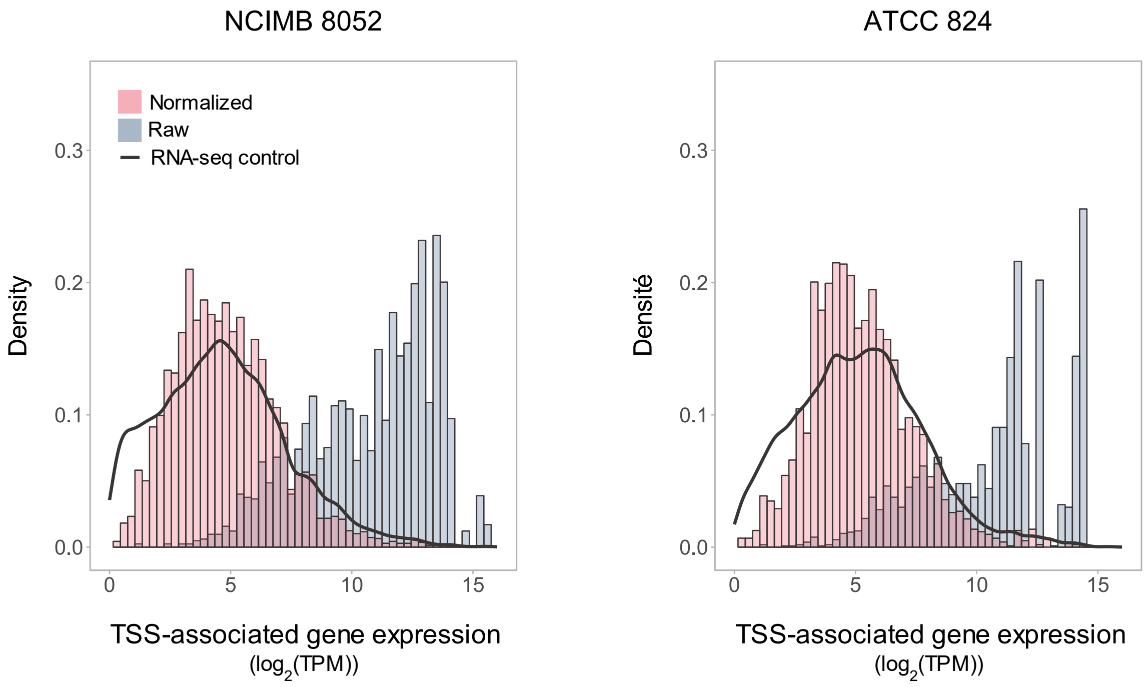

Supplementary Fig S2: **TSS expression distribution with (normalized) and without (raw) expression normalization in *C. beijerinckii* NCIMB 8052 and *C. acetobutylicum* ATCC 824.** 10 RPM TSS detection threshold is shown.

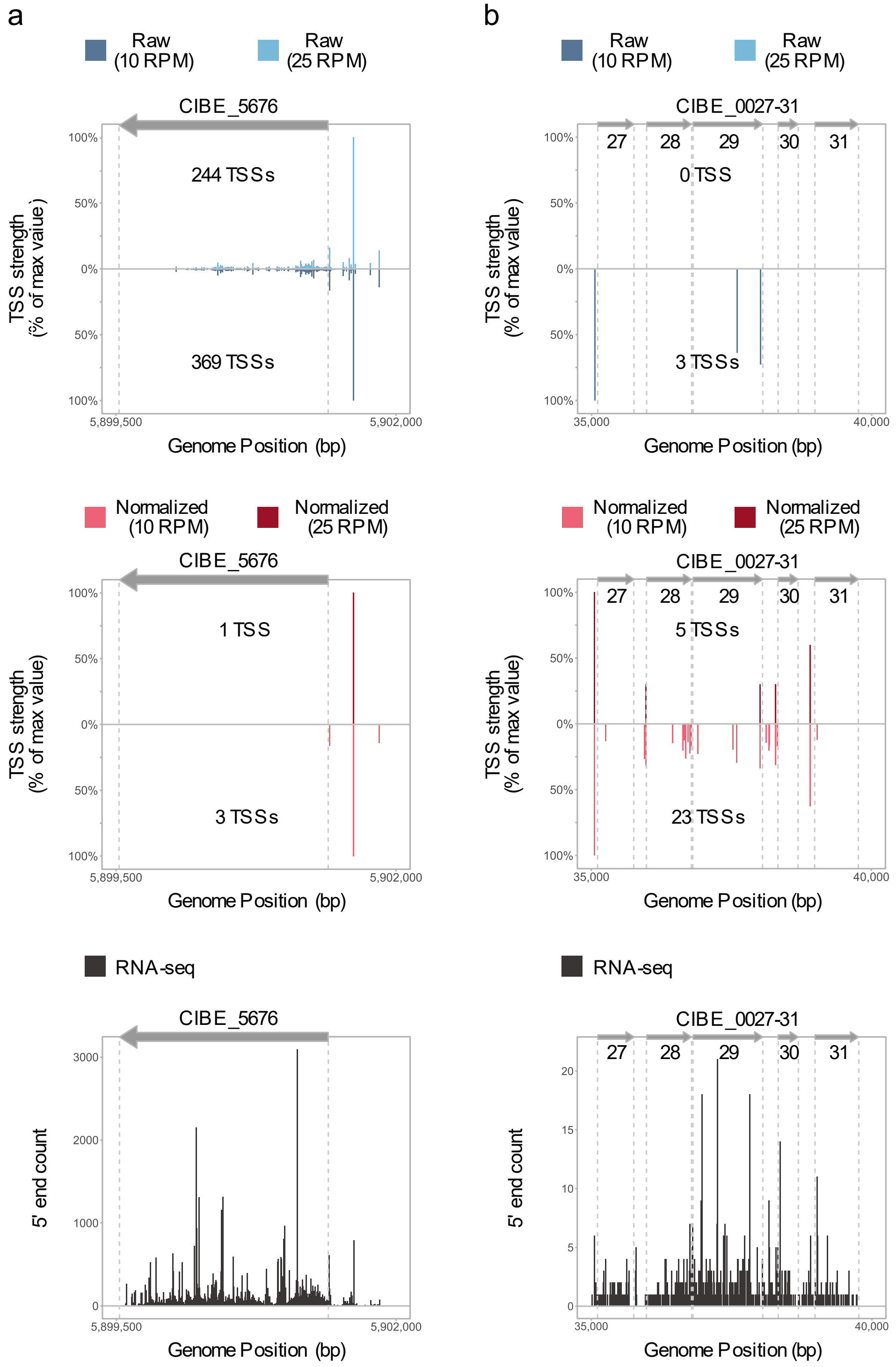

Supplementary Fig S3: **TSSs detected with (normalized) and without (raw) expression normalization at two different genome locations in *C. beijerinckii* DSM 6423.** a. Case of a highly expressed gene (CIBE_5676). b. Case of a group of lowly expressed genes (CIBE_0027 to CIBE_0031).

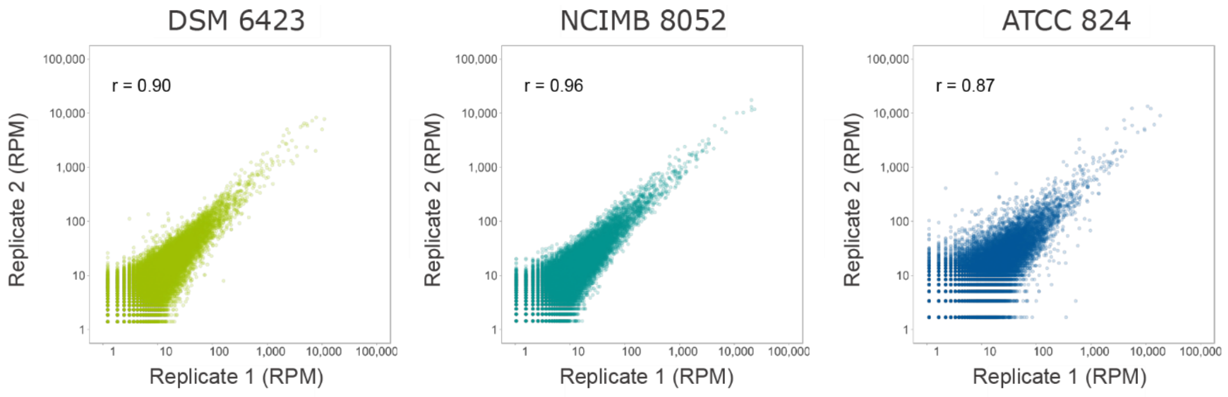

Supplementary Fig S4: **Duplicate analysis for each of the three strains.**

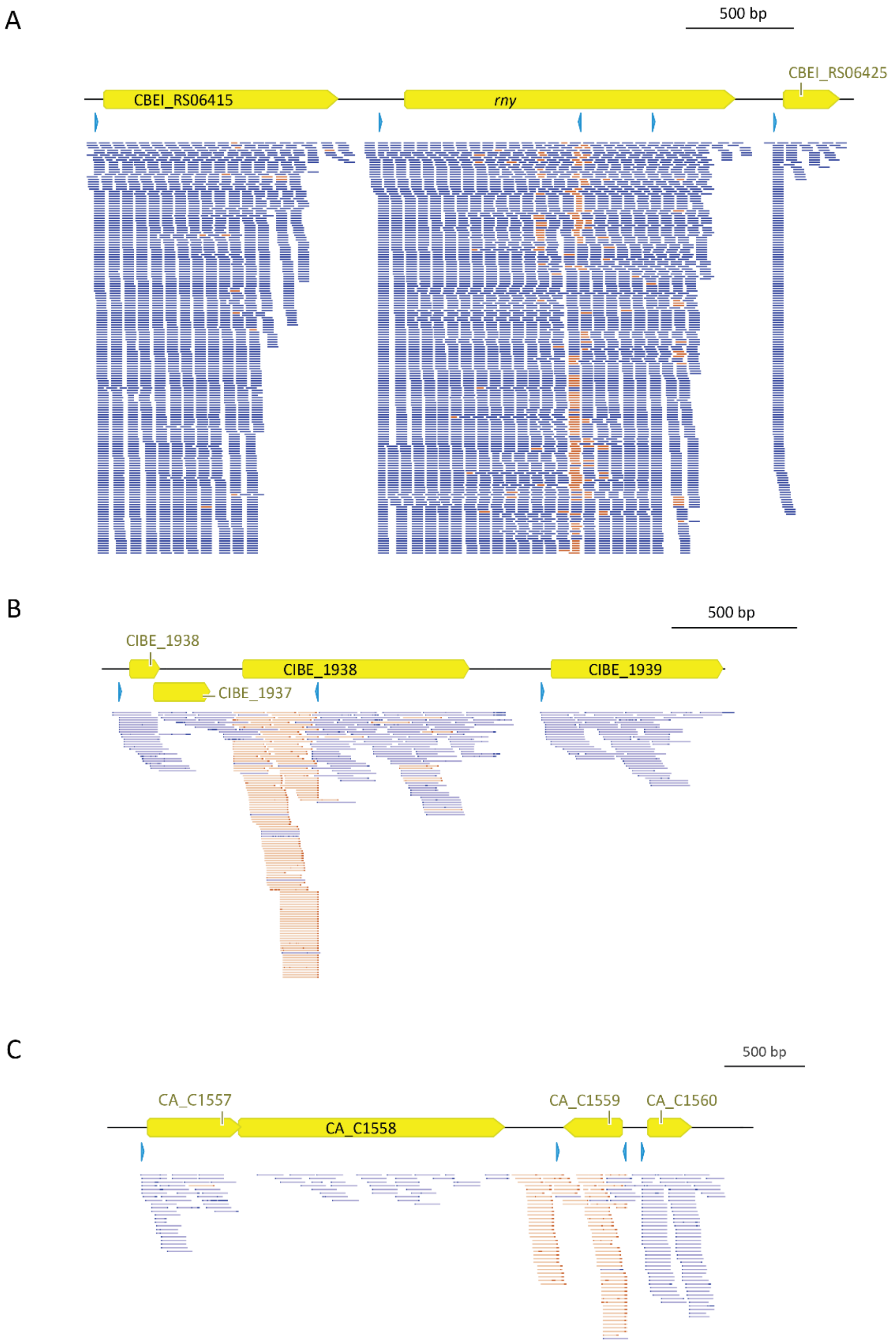

Supplementary Fig S5: **Antisense transcription from antisense TSS.** (A) *rny*, which encodes the ribonuclease Y in *C. beijerinckii* NCIMB 8052, (B) CIBE_1938, which encodes a transposase in *C. beijerinckii* DSM 6423, and (C) CA_C1559, which encodes a transcriptional regulator in *C. acetobutylicum* ATCC 824. Reads in the 5'-3' sense are figured in blue, reads in the 3'-5' sense are figured in orange.

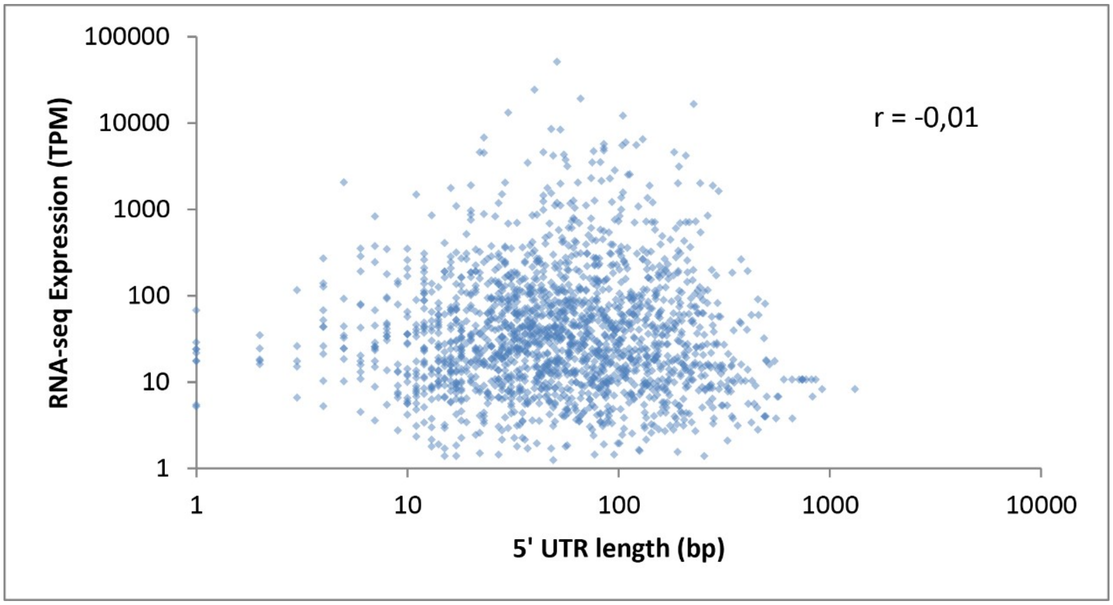

Supplementary Fig S6: **5'UTR length relationship to RNA-seq expression value in *C. beijerinckii* DSM 6423**.

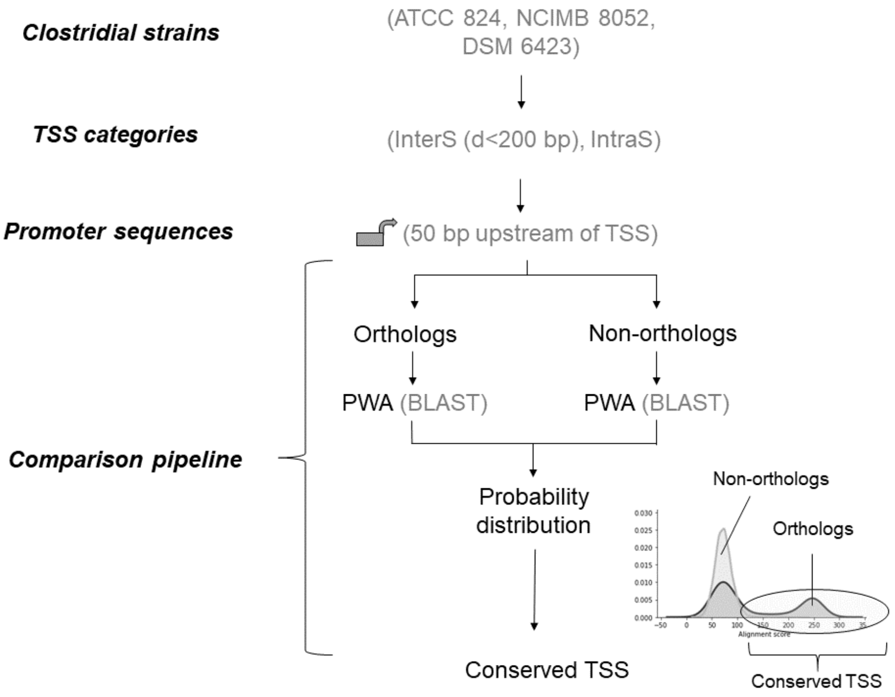

Supplementary Fig S7: **TSS comparison pipeline**. TSS classes InterS (<200 bp from gene start subset) and IntraS were considered. Promoter sequences were first extracted from the genome (50 bp upstream each TSS). All possible pairs were aligned. TSS-associated gene information was used to separate alignments in two categories (alignments associated with orthologous or non-orthologous genes). Distributions of alignment scores were next compared to choose a threshold value discriminating conserved promoters.

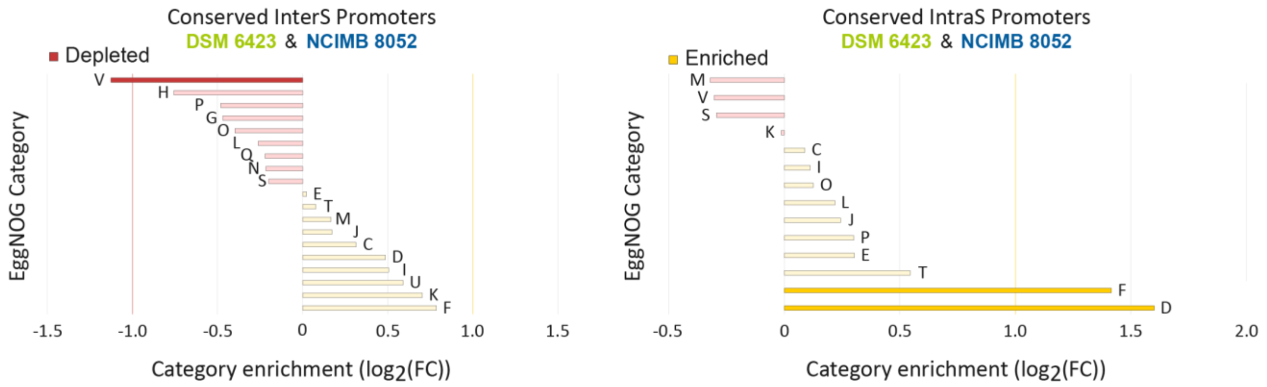

index	Category
F	Nucleotide transport and metabolism
K	Transcription
U	Intracellular trafficking, secretion, and vesicular transport
I	Lipid transport and metabolism
D	Cell cycle control, cell division, chromosome partitioning
C	Energy production and conversion
J	Translation, ribosomal structure and biogenesis
M	Cell wall/membrane/envelope biogenesis
T	Signal transduction mechanisms
E	Amino acid transport and metabolism
S	Function unknown
N	Cell motility
Q	Secondary metabolites biosynthesis, transport and catabolism
L	Replication, recombination and repair
O	Posttranslational modification, protein turnover, chaperones
G	Carbohydrate transport and metabolism
P	Inorganic ion transport and metabolism
H	Coenzyme transport and metabolism
V	Defense mechanisms

Supplementary Fig S8: **Functional enrichment of genes with conserved promoters in C. beijerinckii DSM 6423 and NCIMB 8052.**
